# KRICT-9 inhibits neuroinflammation, amyloidogenesis and memory loss in Alzheimer’s disease models

**DOI:** 10.18632/oncotarget.19818

**Published:** 2017-08-02

**Authors:** Do Yeon Lee, Chul Ju Hwang, Ji Yeon Choi, Mi Hee Park, Min Ji Song, Ki Wan Oh, Sang Bae Han, Woo Kyu Park, Hee Yeong Cho, Sung Yun Cho, Hye Byn Park, Min Jong Song, Jin Tae Hong

**Affiliations:** ^1^ College of Pharmacy and Medical Research Center, Chungbuk National University, Heungduk-gu, Chungbuk 361-951, Republic of Korea; ^2^ Korea Research Institute of Chemical Technology, Yuseong-gu, Daejeon 34114, Republic of Korea; ^3^ Department of Obstetrics and Gynecology, Daejeon St. Mary’s Hospital, College of Medicine, The Catholic University of Korea, Jung-gu, Daejeon 301-723, Republic of Korea

**Keywords:** Alzheimer’s disease, STAT3, neuroinflammation, amyloidogenesis, KRICT-9

## Abstract

Alzheimer’s disease (AD) is one of the most common forms of dementia and is characterized by neuroinflammation and amyloidogenesis. Here we investigated the effects of KRICT-9 on neuroinflammation and amyloidogenesis in *in vitro* and *in vivo* AD models. We found that KRICT-9 decreased lipopolysaccharide (LPS)-induced inflammation in microglial BV-2 cells and astrocytes while reducing nitric oxide generation and expression of inflammatory marker proteins (iNOS and COX-2) as well as APP, BACE1, C99, Iba-1, and GFAP. KRICT-9 also inhibited β-secretase. Pull-down assays and docking model analyses indicated that KRICT-9 binds to the DNA binding domain of signal transducer and activator of transcription 3 (STAT3). KRICT-9 also decreased β-secretase activity and Aβ levels in tissues from LPS-induced mice brains, and it reversed memory impairment in mice. These experiments demonstrated that KRICT-9 protects against LPS-induced neuroinflammation and amyloidogenesis by inhibiting STAT3 activity. This suggests KRICT-9 or KRICT-9-inspired reagents could be used as therapeutic agents to treat AD.

## INTRODUCTION

Alzheimer’s disease (AD) is a neurodegenerative disease characterized by progressive memory loss and other neuropsychiatric symptoms [1, 2]. The main pathological hallmark of AD is the accumulation of amyloid β (Aβ) peptide in the brain [3, 4]. The Aβ peptide is a product of amyloid precursor protein (APP) cleavage by a protease β-secretase (BACE1) [5, 6]. Indeed, the levels of BACE1 and its products (*e.g.,* the C-terminal fragment of APP) are increased in AD brains [7].

AD patients suffer from neuroinflammation, characterized by activation of microglia and astrocytes, with synaptic and neuronal loss resulting in cognitive decline [8]. Microglia are cerebral macrophages that might contribute to neuroinflammation by releasing proinflammatory cytokines [9], which accelerate amyloidogenesis via upregulation of β-secretase in microglia [10]. Furthermore, increased secretion of Aβ upon lipopolysaccharide (LPS) stimulation regulates intracellular APP expression [11]. Also, IFN-γ and TNF-α elevate Aβ plaque deposition and BACE1 expression in microglia [12]. Astrocytes have been also shown to promote β-amyloid clearance and degradation [13]. Astrocytes are often activated by neuroinflammation, leading to astrogliosis. In AD brains, the presence of astrocytes in the cortical molecular layer correlates with amyloid plaques being present in the underlying pyramidal cell layers that accumulate Aβ [14]. It has been proposed that astrocytes can phagocytose Aβ; however, some studies show cases in which astrocytes fail to uptake Aβ from the extracellular space. Moreover, overexpression of APP might help astrocytes to synthesize Aβ [15]. These data suggest that neuroinflammation might be associated with amyloidogenesis stemming from activation of microglia and astrocytes.

We previously reported that LPS treatment induced memory dysfunction by upregulating neuroinflammation and amyloidogenesis in neurons [16–18]. Indeed, LPS can trigger inflammatory cells, such as brain astrocytes and microglia [16]. Intraperitoneal (i.p.) administration of LPS can cause an immediate, strong and persistent upregulation of proinflammatory cytokines such as IL-1β, IL-6, and TNF-α, thereby stimulating amyloidogenesis [10].

STAT3 and p-STAT3 are highly expressed in the basal forebrain, the hippocampus, and the cerebellum in transgenic AD mice [19]. Furthermore, *in vitro* and *in vivo* studies have shown that activation of STAT3 is necessary to trigger a number of inflammatory responses in AD [20, 21]. STAT3 also promotes microglia and astrocyte activation, thus contributing to amyloidogenesis. STAT3 is also necessary for BACE1 transcription upregulation and might thus promote neuroinflammation associated with amyloidogenesis [22]. Many STAT3 inhibitors such as anatabine, Schizandrin A, and Aspirin-triggered Lipoxin A4 (ATL) hinder neuroinflammation and amyloidogenesis [23–25]. We have reported several STAT3 inhibitors such as ent-Sauchinone, 2,4-bis(4-hydroxyphenyl)-2-butenal, and tricin 4′-O-(threo-β-guaiacylglyceryl) ether that inhibit neuroinflammation and amyloidogenesis, and improve memory [7, 26, 27].

Some aminopyridines with substituted hydroxy-benzoxazoles, such as KRICT-9, potently inhibit several kinases [28]. KRICT-9 was initially identified as a hit from the chemical library of Korea Chemical Bank in Korea. Our screenings revealed KRICT-9 as a strong STAT3 luciferase inhibitor. Here, we carried out *in vitro* and *in vivo* experiments using an LPS-induced neuroinflammatory animal model to investigate the anti-neuroinflammatory and anti-amyloidogenic effects of KRICT-9, as well as its ability to promote memory recovery.

## RESULTS

### Effect of KRICT-9 on cell viability, STAT3 transcriptional activity, NO generation, and expression of iNOS and COX-2 as well as Aβ level

Treatment of Raw 264.7 cells, microglial BV-2 cells and astrocytes with KRICT-9 (Supplementary Figure 1) resulted in more than 80% cell viability at concentrations up to 5 μM (Supplementary Figure 2). Since STAT3 promotes amyloidogenesis and neuroinflammation, we first determined STAT3 transcriptional activation in RAW 264.7 cells transiently transfected with STAT3 plasmid. KRICT-9 inhibited LPS-induced STAT3 luciferase activity in a concentration dependent manner (Supplementary Figure 3A). These inhibitory effects were associated with inhibitory effects of KRICT-9 on NO generation (Supplementary Figure 3B).

### KRICT-9 inhibits LPS-induced NO production as well as COX-2 and iNOS expression in BV-2 cells and astrocytes

**Figure 1:**
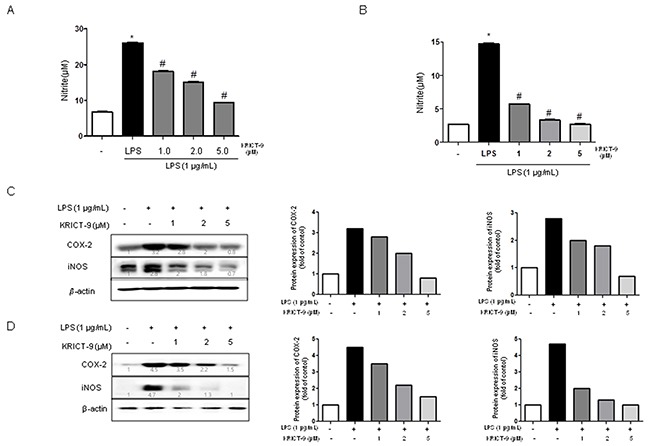
Effect of KRICT-9 on LPS-induced NO release and on protein expression of iNOS and COX-2 in microglial BV-2 cells and astrocytes Cells were treated with 1 μg/mL of LPS alone, or with LPS plus different concentrations (1, 2, 5 μM) of KRICT-9, at 37°C for 24 h. NO levels were determined by Griess reaction as described in Methods, in supernatants from **(A)** microglial BV-2 cells and, **(B)** astrocytes. Equal amounts of total protein (20 μg/lane) were subjected to 8% SDS-PAGE, and the expression of iNOS and COX-2 were detected by western blotting using specific antibodies in **(C)** microglia, and **(D)** astrocytes. Quantitative analysis of the western band was performed using ImageJ program. β-actin was used here as an internal control. Values represent means ±SD for three independent experiments performed in triplicate. * indicates significantly different from the control group (p<0.05). # indicates significantly different from the LPS treated group (P < 0.05).

STAT3 promotes iNOS and COX-2 expression, thereby contributing to neuroinflammation. We used Griess assay to investigate the effect of KRICT-9 on LPS-induced NO production in astrocytes and microglial BV-2 cells. LPS treatment (1 μg/mL) elevated NO levels in microglia and astrocytes. On the contrary, LPS-induced NO production was decreased by KRICT-9 in microglial BV-2 cells (Figure 1A) and astrocytes (Figure 1B). To determine whether KRICT-9 suppresses the expression of inflammatory genes, we investigated COX-2 and iNOS expression using western blot. The expression of COX-2 and iNOS protein was increased above the basal level in response to LPS (1 μg/ml) after 24 h. However, co-treatment with KRICT-9 (1, 2, and 5 μM) caused a decrease in the levels of LPS-induced COX-2 and iNOS in microglial BV-2 cells (Figure 1C) and astrocytes (Figure 1D).

### KRICT-9 inhibits STAT3 activity in microglia and astrocytes

**Figure 2:**
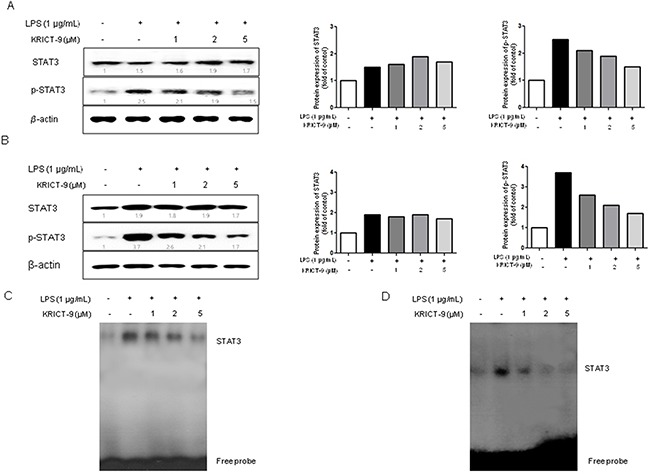
KRICT-9 inhibits LPS-induced STAT3 DNA binding activity in microglia BV-2 cells and astrocytes The cells were treated with 1 μg/mL of LPS alone, or with LPS plus different concentrations (1, 2, 5 μM) of KRICT-9 at 37°C for 1 h. Equal amounts of total proteins (20 μg/lane) were subjected to 8% SDS-PAGE, and activation of STAT3 (phosphorylation) was detected by western blotting using specific antibodies in **(A)** microglial BV-2 cells, and **(B)** astrocytes. Quantitative analysis of the western band was performed using ImageJ program. STAT3 DNA binding activity was determined by EMSA in **(C)** microglial BV-2 cells, and **(D)** astrocytes. Experiments were performed in triplicate.

STAT3 promotes neuroinflammation and amyloido-genesis through the regulation of several genes. To investigate whether KRICT-9 can inhibit LPS-induced STAT3 activity, we treated microglial BV-2 cells and astrocytes with LPS (1 μg/mL) or co-treated them with LPS and KRICT-9 (1, 2 and 5 μM) for 6 h. LPS significantly induced STAT3 phosphorylation. On the other hand, LPS-induced STAT3 phosphorylation was inhibited by co-treatment with KRICT-9 in microglial BV-2 cells (Figure 2A) and astrocytes (Figure 2B) in a concentration dependent manner. Consistent with such effect, the DNA binding activity of STAT3 elevated by LPS was also reduced by KRICT-9 in microglial BV-2 cells (Figure 2C) and astrocytes (Figure 2D).

### Interaction between KRICT-9 and STAT3

**Figure 3:**
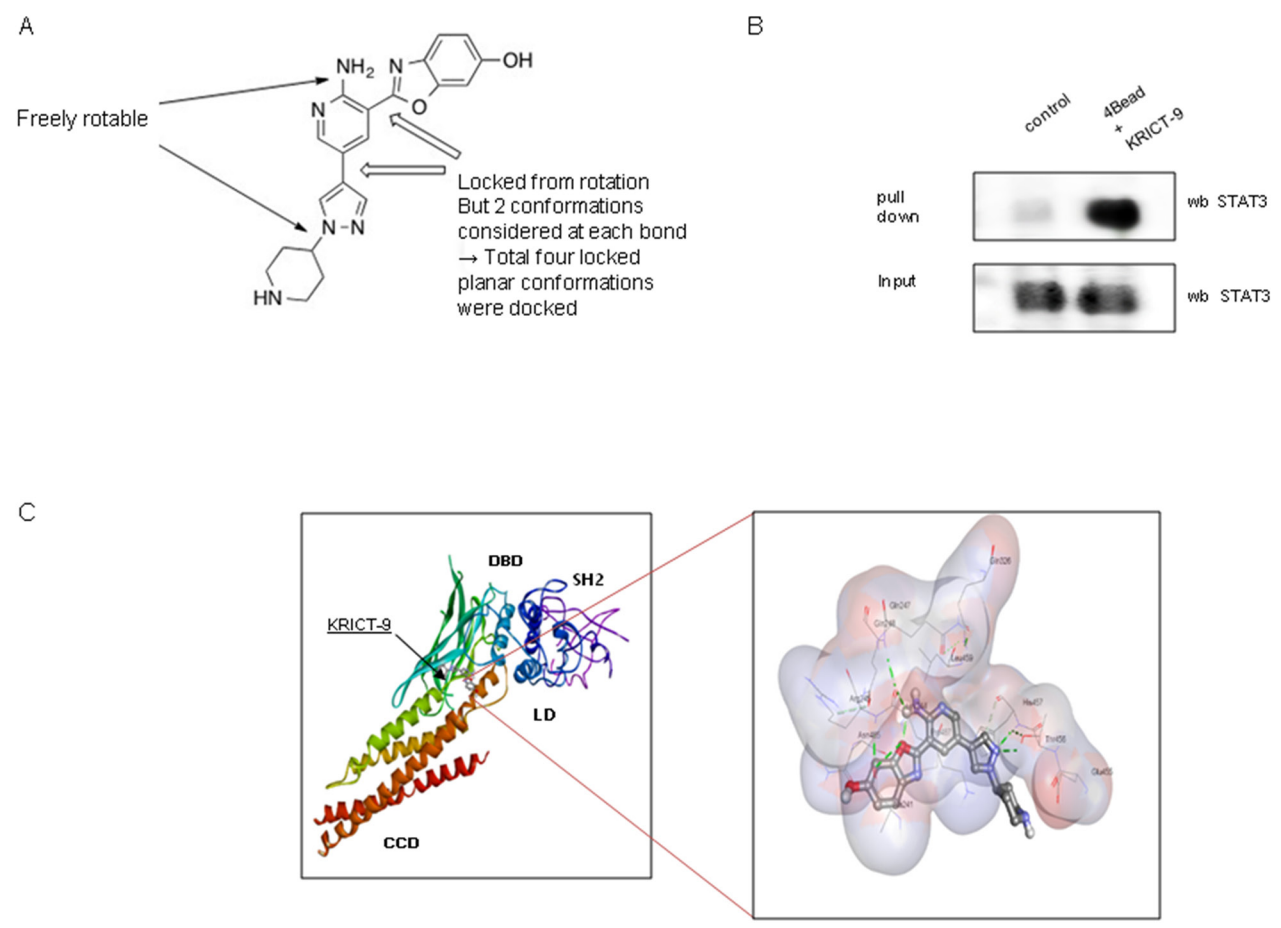
**(A)** Chemical structure of KRICT-9. **(B)** Pull-down assay showing interaction between the KRICT-9 and STAT3. KRICT-9 was conjugated with KRICT-9-activated Sepharose 4B. **(C)** Docking model for KRICT-9 interacting with STAT3.

We also investigated whether KRICT-9’s inhibited STAT3 by directly binding to it. The possible binding site is indicated in Figure 3A. We performed pull-down assays using KRICT-9-sepharose 4B beads. Binding of KRICT-9 to STAT3 was then test for by immunoblotting with STAT3 antibody. We found that KRICT-9 interacted with STAT3 in lysates from microglial BV-2 cells and with recombinant STAT3 (Figure 3B). We also carried out computational docking analyses to pinpoint the binding site of KRICT-9 on STAT3 using Autodock Vina software. Our results showed that KRICT-9 binds to the following residues on STAT3: Ala241, Lys244, Arg245, Gln247, Gln248, Gln326, Glu455, Thr456, His457, Leu459, Asn485, & Pro487) (Figure 3C).

### KRICT-9 inhibits amyloidogenesis in astrocytes and microglia cells

**Figure 4:**
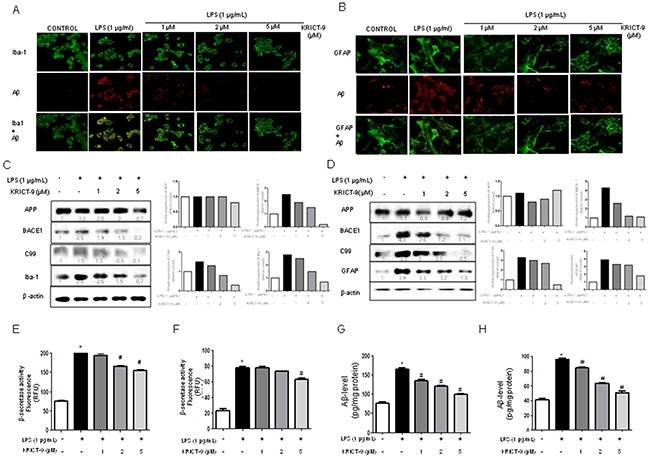
Effect of KRICT-9 on the levels of APP, BACE1, C99, lba-1, Aβ_42_, and GFAP, as well as on β-secretase activity Microglial BV-2 cells were incubated with anti-IBA-1 (green) and anti- Aβ_1−42_ (red) primary antibodies and the cultured astrocytes were incubated with anti-GFAP (green) and anti- Aβ_1−42_ (red) primary antibodies. **(A-B)** The effect of KRICT-9 on fluorescence was detected using Alexa 488-conjugated anti-mouse/goat and Alexa 568-conjugated anti-rabbit secondary antibodies. Expressions of APP, BACE1, C99, lba-1, and GFAP as detected by western blot using specific antibodies in **(C)** microglial BV-2 cells, and (D) astrocytes. Quantitative analysis of the western band was performed using ImageJ. Each blot is representative of three experiments. β-actin was used as an internal control. *p < 0.05 indicates significantly different from the LPS-treated group. Co-treatments with KRICT-9 and LPS for 24 h were used. **(E-F)** β-secretase activity. Cells were collected to determine Aβ_42_ by ELISA from **(G)** microglial BV-2 cells, and **(H)** astrocytes. Values represent means ±SD for three independent experiments performed. *p <0.05 compared to control, #p < 0.05 compared LPS.

The activation of microglia and astrocytes elevates neuroinflammation and leads to amyloidogenesis. Therefore, we investigated whether the amount of GFAP-positive astrocytes and Aβ accumulation increase simultaneously upon LPS treatment, and whether KRICT-9 reduces astrocyte activation and Aβ accumulation. Cells immunoreactive to GFAP and accumulating Aβ were analyzed using immunofluorescence. The number of co-reactive cells for both markers was elevated by LPS treatment, but was decreased by treatment with KRICT-9 (Figure 4B). Microglial BV-2 cells were also used for further studies. The number of cells positive for Aβ accumulation (Aβ_1-42_- positive cells) in microglia (Iba1-positive cells) also increased upon LPS treatment, and also decreased after treatment with KRICT-9 (Figure 4A). We next investigated whether KRICT-9 reduced amyloidogenesis induced by LPS. Unstimulated microglia and astrocytes showed lower levels of APP, β-site APP cleavage enzyme (BACE1), C99, Iba-1, and GFAP protein (Figure 4C, 4D). On the other hand, the expressions of APP, BACE, C99, Iba-1, and GFAP proteins increased in response to LPS (1 mg/ml) after 24 h. However, KRICT-9 inhibited the LPS-induced expression of APP, BACE1, and C99 in a concentration-dependent manner. LPS-induced β-secretase activity was also inhibited in BV-2 cells (Figure 4E) and astrocytes (Figure 4F). KRICT-9 treatment also decreased LPS-induced Aβ-levels in microglial BV-2 cells (Figure 4G) and astrocytes (Figure 4H). These results show that KRICT-9 inhibits inflammation and amyloidogenesis *in vitro*.

### STAT3’s involvement in KRICT-9’s inhibition of LPS-induced neuroinflammation and amyloidogenesis

**Figure 5:**
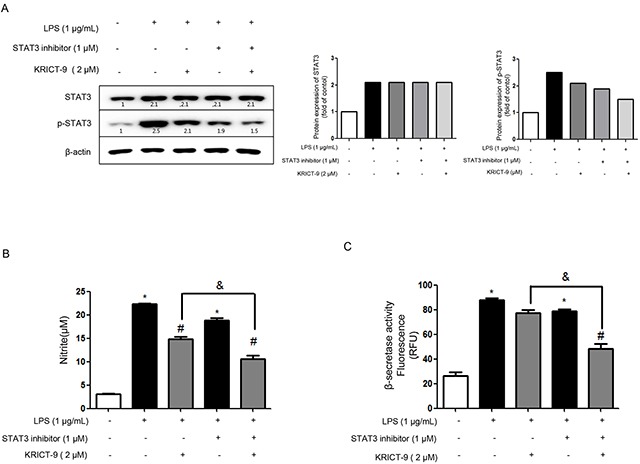
Effects of STAT3 inhibitor and KRICT-9 in astrocytes **(A)** Western blot showing activation of STAT3 (phosphorylation) using specific antibodies in astrocytes (β-actin was used an internal control). Plots showing **(B)** NO levels, and **(C)** β-secretase activity. Values are presented as mean ± S.D. for three independent experiments performed in triplicate. *p <0.05 compared to control, #p < 0.05 compared LPS, &p < 0.05.

We also investigated the involvement of the STAT3 pathway in KRICT-9’s inhibition of neuroinflammation and amyloidogenesis. To this end,, we co-treated cells with KRICT-9 and a static STAT3 inhibitor and then measured STAT3, p-STAT3, and NO release. The combination of KRICT-9 (2 μM) and STAT3 inhibitor (1 μM) inhibited STAT3 phosphorylation compared to KRICT-9 or STAT3 inhibitor treatment alone (Figure 5A). Treatment with KRICT-9 (2 μM) decreased NO generation (33.59% inhibition) and β-secretase activity (11.61%) in cultured astrocytes. However, the inhibitory effect of KRICT-9 on NO generation (44.96%) and β-secretase (37.97%) was augmented by co-treatment with STAT3 inhibitor in cultured astrocytes.

### KRICT-9 treatment inhibits memory impairment in LPS-treated mice

**Figure 6:**
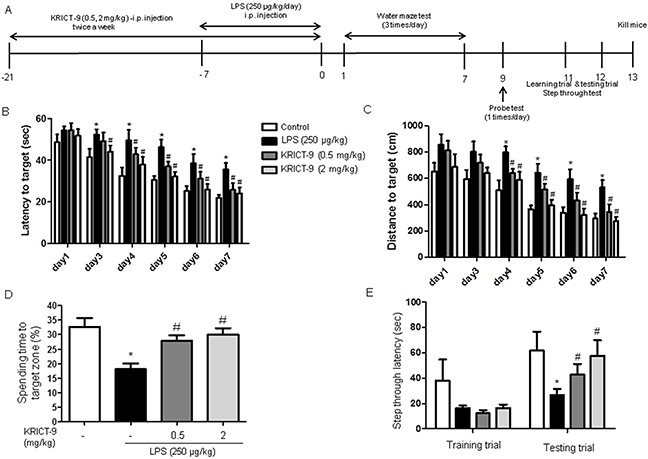
KRICT-9 improves memory in LPS-treated mice **(A)** Timeline depicting KRICT-9 treatment and assessment of cognitive functions. Arrowheads represent days on which acquisition tests were conducted. Mice were treated with KRICT-9 (0.5 and 2 mg/kg, i.p.) 1 h after LPS treatment (250 μg/kg, i.p.) for 3 weeks. Morris water maze and passive avoidance tests show that LPS injection elongates **(B)** escape time and **(C)** distance. **(D)** Figure shows decreased spending time to target zone for LPS-treated mice. **(E)** Plot showing that the memory deficit induced by LPS was attenuated by KRICT-9 treatment. Values are presented as mean ± S.D (n=8). *p < 0.05 compared to control, #p < 0.05 compared to LPS.

We used the water maze and passive avoidance performance tests to assess cognitive impairment and the effect KRICT-9 treatment on memory. We investigated spatial memory in mice by measuring escape latency and distance in the water maze test. LPS-injected mice (35.64±3.07) learned more slowly than control mice (21.86±1.48), and mice treated with 0.5 mg/kg (25.80±3.170) (p=0.03) or 2 mg/kg KRICT-9 (24.00± 2.93) (p=0.01) exhibited a reduction in escape latency over the training period (Figure 6B). Mice treated with 0.5 mg/kg (344.3±59.01) or 2 mg/kg KRICT-9 (272.8±35.16) also exhibited a shorter escape distance (Figure 6C) compared mice treated with LPS (531.4±55.81) (p=0.03). After the final day of the water maze test, we performed a probe test to calculate the time spent in the target quadrant zone, effectively testing for maintenance of memory. Mice treated with 0.5 mg/kg (27.90±1.87) or 2 mg/kg KRICT-9 (30.00±2.21) spent much more time in the quadrant zone than LPS-injected mice (18.13±1.90) (Figure 6D). We further tested for memory maintenance using a passive avoidance test. Although there was no difference in the learning trial, mice treated with 0.5 mg/kg (46.48±7.46) or 2 mg/kg KRICT-9 (57.75±12.28) recorded increased step-through latency compared with LPS treated mice (26.80±4.85) (Figure 6E).

### KRICT-9 prevents LPS-induced amyloidogenesis and accumulation of Aβ

**Figure 7:**
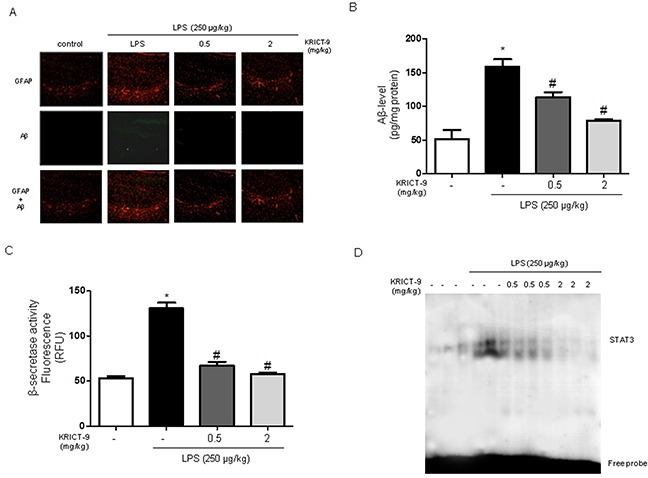
KRICT-9 inhibits accumulation of Aβ_1-42_, β-secretase and STAT3 activity in the brains of LPS-injected mice **(A)** Aβ accumulation in the brains of LPS-injected mice by thioflavin S staining. **(B)** Levels of Aβ_1-42_ in the brains of LPS-injected mice as measured by ELISA. **(C)** Plot showing β-secretase activity in the brains of LPS-injected mice. **(D)** Graph showing STAT3 DNA binding activity in the brains of LPS-injected mice as determined by EMSA. Values are represented as mean ± S.D. (n=5). * Comparison against controls (p<0.05), and # against LPS (p<0.05).

The accumulation of Aβ peptides and neuro-inflammation are hallmarks of AD. Therefore, we investigated whether KRICT-9 reduced LPS-induced neuroinflammation and amyloidogenesis, while promoting memory recovery. GFAP and Aβ were identified using immunofluorescence and thioflavin S staining, which are methods widely used to measure Aβ accumulation. We found higher Aβ accumulation in the brains of LPS-treated mice than in those of KRICT-9-treated mice (Figure 7A). To test whether amyloidogenesis was reduced by KRICT-9, we measured Aβ_1−42_ levels and β-secretase activity in the whole brain. Aβ_1−42_ levels and β -secretase activity were increased in LPS-treated mice, but decreased in KRICT-9-treated mice (Figure 7B and 7C).

### Inhibitory effect of KRICT-9 inhibits LPS-induced STAT3 activation in brain

To determine whether KRICT-9 is capable of suppressing the DNA-binding activity of STAT3 in mice, we measured DNA-binding activity using nuclear extracts from mice brains. STAT3 DNA-binding activity was induced by LPS; however, it was blocked by KRICT-9, which also inhibited phosphorylation of STAT3 (Figure 7D).

## DISCUSSION

Neuroinflammation and amyloidogenesis are pathogenesis hallmarks in AD, leading to impaired memory in AD patients [29]. In our present study, we found that KRICT-9 treatment alleviated LPS-induced memory impairment. Indeed, KRICT-9 ameliorated amyloidogenesis and neuroinflammation in mice brains and in cultured inflammatory cells through direct inhibition of STAT3. Previous studies by our group and others demonstrated that LPS injection causes neuroinflammation and amyloidogenesis, leading to impaired memory [17, 30–32]. In previous studies, we showed that a variety of anti-inflammatory compounds, such as Epigallocatechin-3-gallate, Ethanol extract of Magnolia officinalis, 2,4-bis(4-hydroxyphenyl)-2-butenal, 4-O-methylhonokiol, obovatol, thiacremonone, and bee venom hindered neuroinflammation and amyloidogenesis, thereby improving memory [7, 16, 18, 33]. In agreement with our previous studies, our present study showed that KRICT-9 decreased LPS-induced inflammatory responses (such as NO release and the expression of the inflammatory marker proteins iNOS and COX-2) and amyloidogenesis through inhibition of β-secretase activity and Aβ formation. Overactive astrocytes and microglia cells can cause neuroinflammation. Lee *et al.* discovered that LPS-activated astrocytes and microglial BV-2 cells show high levels of TNF-α, IL-1β, iNOS, and COX-2 [34]. Elevated levels of beta APP were also seen in primary cultured astrocytes and microglia cells from APdE9 transgenic mouse brains [35]. Salemme *et al.* showed that amyloidogenesis was inhibited by dihydroasparagusic acid treatment of lipopolysaccharide-activated microglial cells [36]. Thus, we hypothesized that KRICT-9’s inactivation of astrocytes and microglia cells and inhibition of LPS-induced neuroinflammation reduces amyloidogenesis. Neuroinflammation in response to surgery causes postoperative cognitive dysfunction through gliosis, microgliosis, and astrogliosis, all of which increase production of Aβ in elderly patients [37]. Thus, we also hypothesized that KRICT-9 treatment could lead to memory recovery in AD.

STAT3 activates the transcription of BACE1, APP, and β-secretase, thereby increasing Aβ production [38]. Indeed, downregulation of STAT3 activates astrocytes and microglia and increases BACE1 transcription [39]. The activated astrocytes and microglia correlates with the presence of amyloid plaques in AD. Moreover, STAT3 phosphorylation is increased in the brain of AD patients and Aβ peptides have been shown to induce STAT3 phosphorylation, leading to neurodegeneration [23]. LPS-induced STAT3 phosphorylation was also inhibited by KRICT-9 in microglial BV-2 cells and astrocytes as well as in mice brain in a concentration-dependent manner. One recent study revealed that consistent activation of astroglial STAT3 following systemic LPS treatment (10 mg/kg) correlated with brain cell and microvasculature injury in the hippocampus, resulting in cognitive impairment [40]. Therefore, we hypothesized that KRICT-9 might target STAT3. Indeed, here our docking models and pull down assays suggest that KRICT-9 directly binds to the DNA binding domain of STAT3. Furthermore, in agreement with these data, we showed that KRICT-9 inhibits STAT3 activity. Moreover, the combination of KRICT-9 and STAT3 inhibitor showed increased suppression of neuroinflammation and amyloidogenesis. Similar effects have been shown for other compounds [7, 26, 41, 42].

Moreover, several metrics such as the CMC-like and WDI-like rules as well as Caco2 cell permeability and plasma protein binding highlight KRICT-9 as a viable drug. Indeed, KRICT-9 is negative for all predicted toxicities evaluated by computational ADME QSAR models using preAPMET (http://preadmet.bmdrc.org) and StarDrop (http://www.optibrium.com). Thus, our results here warrant running clinical trials to test KRICT-9 as a therapeutic agent to treat patients with AD.

## MATERIALS AND METHODS

### Materials

2-(2-Amino-5-(1-(piperidin-4-yl)-1*H*-pyrazol-3-yl)pyridin-3-yl)benzo[d]oxazol-6-ol was synthesized from *tert*-butyl 4-(4-(6-(*tert*-butylamino)-5-(6-chlorobenzo[*d*]oxazol-2-yl)pyridin-3-yl)-1*H*-pyrazol-1-yl)piperidine-1-carboxylate in three steps, palladium-catalyzed cross-coupling reaction, hydroxylation, and deprotection of protecting groups [28]. The structure of KRICT-9 is shown in Supplementary Figure 1A. The ^1^H NMR figure is shown in Supplementary Figure 1B.

### Chemicals and reagents

LPS (from Escherichia coli 055:B5) was obtained from Sigma Aldrich (St Louis, MO). Dulbecco’s modified Eagle’s medium (DMEM), fetal bovine serum, penicillin and streptomycin were purchased from Invitrogen (Carlsbad, CA).

### Ethical approval

This study was performed according to the guidelines for animal experiments of the Faculty of the Disease Animal Model Research Center at the Korea Research Institute of Bioscience and Biotechnology (Daejeon, Korea) as well as the Institutional Animal Care and Use Committee (IACUC) of the Laboratory Animal Research Center at Chungbuk National University, Korea (CBNUA-929-16-01). All efforts were made to minimize animal suffering, and to reduce the number of animals used. The mice were housed in a temperature-controlled room (21-25°C), with relative humidity ranging from 45 to 65%, and following a light-dark cycle. All studies were approved by and performed according to the ethical guidelines by the Chungbuk National University Animal Care Committee (CBNUA-929-16-01).

### *In vivo* experimental design

Eight week old male imprinting control region (ICR) mice (Daehan Biolink, Chungcheongbuk-do, Korea) were used in observance with KFDA guidelines. In order to establish a neuroinflammatory cognitive impairment model, intraperitoneal LPS treatment (0.25 mg/kg) was administered [18]. Mice were divided into four groups: (I) Control group, (II) LPS group, (III) KRICT-9 0.5 mg/kg + LPS group, and (IV) KRICT-9 2 mg/kg + LPS group, with 10 mice each. KRICT-9 was given to groups III and IV injected intraperitoneally at a dose of 0.5 mg/kg and 2 mg/kg two times a week for three weeks. Intraperitoneal (i.p.) injection of LPS (250 μg/kg) was administered (except to the control group) on the 3rd week for seven days. Behavior, learning, and memory were then assessed using three tests (water maze, probe, and passive avoidance test). The water maze test was performed on even days after KRICT-9 and LPS administration. The probe test was performed one day after the water maze test. The passive avoidance test was performed one day after the probe test.

### Morris water maze

The water maze test is commonly used to assess memory. We strictly followed it as described by Morris et al. [43]. The dSMART-CS (Panlab, Barcelona, Spain) program and equipment were used. We used a circular plastic pool (height: 35 cm, diameter: 100 cm) full with squid ink water and kept it at 22-25°C. An escape platform (height: 14.5 cm, diameter: 4.5 cm) was immersed 1-1.5 cm below the surface of the water. On training trials, when firstly placed in the pool of water, the mice then remain on the platform for 2 min. After that, they returned to their cage. The mice that could not find the platform within 1 min were put on the platform for 10 s at the end of the trial. When mice stayed on the platform for 3 s, we let them remain on the platform for 7 s. These trials were carried out by a single platform and two starting positions of rotational starts. Connecting with SMART-LD (Panlab, Barcelona, Spain), a camera above the center of the pool was used to monitor the escape latency and escape distance of each mouse.

### Probe test

When the water maze test was performed, a probe test was carried out 24h after to assess memory consolidation. After the platform used for the water maze test was removed, we let the mice swim freely. During 60 s, the swimming pattern of each mouse was recorded using SMART-LD. The time they stayed at the target quadrant area was measured to figure out consolidated spatial memory.

### Passive avoidance performance test

The passive avoidance test is widely accepted as a simple memory test. Its response was determined using a “step-through” apparatus (Med Associates Inc, Vermont, USA) that consists of an illuminated dark compartment (each 20.3 × 15.9 × 21.3 cm) adjoining each other via a small gate with a grid floor of 3.175 mm stainless steel rods set 8 mm apart. At first, the mice were placed in the illuminated compartment facing away from the dark compartment for the training trial. When the mice moved completely into the dark compartment, they were given an electric shock (3 mA for 3 s) and returned to their cage. After day one, the mice were positioned in the illuminated compartment and the latency period was measured (the latency period is when the mice are in the dark compartment called “retention”). We also recorded the step-through latency, which is the time at which the mice entered the dark compartment.

### Brain collection and preservation

After behavioral tests, mice were infused with phosphate-buffered saline (PBS, pH 7.4) and heparin under inhaled CO2 anesthetization. Brains were immediately excised and sectioned into two parts. One part was stored at −80°C while the other was fixed in 4 % paraformaldehyde for 72 h at 4°C and transferred to a 30 % sucrose solution.

### Astrocytes and microglial BV-2 cell culture and transfection

Astrocytes were obtained from the cerebral cortex of 3-day-old neonatal rats as previously described with slight modifications [44]. The cerebral cortex was divided into a single-cell suspension by trypsin and mechanical disruption. Cells were seeded with PLL (0.1 mg/ml, Sigma)-coated culture flasks and Dulbecco’s modified eagle medium (DMEM)/F-12 (Invitrogen, Carlsbad, CA) containing 5% fetal bovine serum (FBS) (Invitrogen). The medium was replaced after 24 h. From then, the medium was changed every three days. After a 10-12 day incubation during which cells became confluent, loosely attached microglia and precursor cells of oligodendrocytes were removed from the cell monolayer. Astrocytes were subsequently detached using trypsin-EDTA and put into PLL-coated 8-well plates. The probability of glial fibrillary acidic protein (GFAP) positive cells in our culture system was over 95%. Primary astrocyte cultures were treated with LPS (1 μg/ml). Microglial BV-2 cells were cultured in DMEM supplemented with FBS (10%), NaHCO3 (40 mM), penicillin (100 units/ml, Invitrogen), and streptomycin (100 mg/ml, Invitrogen) at 37°C in an atmosphere of 5% CO2. The cells were supplemented with LPS (1 μg/ml)-induced KRICT-9 at 1, 2, and 5 μM for 24 h and transiently transfected with STAT3 siRNA (Santa Cruz Biotechnology, Santa Cruz, CA) using the WelFect-EX PLUS reagent in OPTI-MEN, according to the manufacturer’s specification (WelGENE, Seoul, Korea).

### Cell viability

To investigate cell viability, cells were seeded in 100 mm plates (2×10^6^ cells/well) and treated with different concentrations of KRICT-9 (1, 2, and 5 μM) in the presence and/or absence of LPS (1 μg/ml) for 24 h. The cells were then detached using trypsin. The pellet was collected after 5 min centrifugation at 1,500 rpm and was resuspended in 10 ml of PBS. Subsequently, 0.1 ml of 0.2% trypan blue (Sigma) were added to the cell suspension in each solution (0.9 ml each). A drop of suspension was placed into a Neubauer chamber to count the number of living cells. Stained cells were considered to be dead. Each assay was performed in triplicate.

### Nitric oxide measurement

The concentrations of KRICT-9 in LPS (1 μg/ml)-exposed astrocytes and microglial BV-2 cells were 1, 2, 5 μM for 24 h. The positive group was treated with LPS only. The cultured medium was infused with Griess reagent [0.1% N-(1-naphthyl)-ethylenediamine, 1% sulfanilamide in 5% phosphoric acid] (Sigma) and incubated at room temperature for 10 min. The absorbance at 540 nm was measured in a microplate reader (Molecular Devices).

### Thioflavin S staining

When transferred to a 30% sucrose solution, the brains were cut into 20 μm sections using a cryostat microtome (Leica CM 1850; Leica Microsystems, Seoul, Korea). The brains were washed in distilled water for 5 min, transferred to gelatin-coated slices, and stained with in 1% thioflavin S for 5 min. Subsequently, brain sections were washed in distilled water and dehydrated with 50, 70, 90, and 100% ethanol for 2 min. The sections were then mounted in medium (Fluoromount™ Aqueous Mounting Medium, Sigma, St Louis, MO, USA) and visualized with a fluorescence microscope (Axio Observer A1, Carl Zeiss, Oberkochen, Germany) (× 100).

### Luciferase activity

RAW 264.7 cells were plated on 12-well plates (1 × 10^5^ cells / well) and transiently transfected with STAT3-Luc plasmid construct using a mixture of plasmid and Lipofectamine 3000 for 24 h according to the manufacturer’s specifications (Invitrogen, Carlsbad, CA, USA) [45, 46]. The transfected cells were treated with 1, 2, and 5 μM of KRICT-9 for 24 h. Luciferase activity was assessed using a luciferase assay kit (Promega, Madison, USA), and the results were read on a luminometer (WinGlow, Bad Wildbad, Germany).

### Fluorescence microscopy

Fixed cells were exposed to the following primary antibodies: GFAP (Cell Signaling Technology), Aβ (Novus Biologicals, Littleton, CO; NBP2-13075) (1:100 dilutions in blocking serum, Abcam), and Iba1 (1:100 dilution in blocking serum, Wako) at room temperature for 1 h. The cells were then washed twice with ice PBS and incubated with an anti-rabbit or mouse secondary antibody conjugated to Alexa Fluor 488 or 568 (Invitrogen-Molecular Probes, Carlsbad, CA) at room temperature for 1 h. Immunofluorescence images were acquired with an inverted fluorescent microscope Zeiss Axiovert 200 M (Carl Zeiss, Thornwood, NY).

### Western blot analysis

Cultured cells and brain tissue were homogenized with lysis buffer (50mM Tris, pH 8.0, 150 mM sodium chloride (NaCl), 0.02% sodiumazide, 0.2% sodium dodecyl sulfate (SDS), 1 mM phenylmethanesulfonyl fluoride (PMSF), 10 μl/ml aprotinin, 1% IGEPAL CA-630, 10 mM sodium fluoride, 0.5 mM EDTA, 0.1 mM ethylene glycol tetraacetic acid, and 0.5% sodium deoxycholate) and centrifuged at 15,000 g for 15 min. The cytosolic and nuclear fractions were extracted from cells and brain tissues using electromobility shift assay (EMSA). Protein (40 μg) from brain tissues or astrocytes was run through an SDS/10-15% polyacrylamide gel and transferred to a nitrocellulose membrane (Hybond ECL, Amersham Pharmacia Biotech). This nitrocellulose membrane was then incubated at room temperature with antibodies: anti-Aβ (D54D2) (1/500, Cell Signaling Technology, Beverly, MA), anti-BACE (1/500, Sigma), anti-C99 and anti-APP (1/500, Sigma, ABR and Covance), and anti β-actin (1/2000, Santa Cruz Biotechnology). COX-2 (1/1000, Cayman Chemical Company, Ann Arbor, MI), iNOS (1/1000, Abcam, Cambridge, UK), STAT3, and p-STAT3(1/1000, Santa Cruz Biotechnology) were used as rabbit polyclonal second antibodies. In addition, the blot was incubated with corresponding conjugated anti-mouse or anti-rabbit antibodies (1/4000, Santa Cruz Biotechnology). After incubation with ECL solution, the membrane was scanned using MyImage (SLB, Seoul, Korea), and quantified using Labworks 4.0 software (UVP Inc., Upland, CA, USA).

### Electro mobility shift assay

EMSA assay was conducted according to the manufacturer’s recommendations (Promega, Madison, WI). Astrocytes were washed twice with 1 × PBS and were spun down at 15,000 rpm for 5 min in a cold Eppendorf tube. Solution A (50 mM HEPES, pH 7.4, 10 mM KCl, 1 Mm EDTA, 1 mM EGTA, 1 mM dithiothreitol, 0.1 μg/ml PMSF, 1 μg/ml pepstatin A, 1 μg/ml leupeptin, 10 μg/ml soybean trypsin inhibitor, 10 μg/ml aprotinin, and 0.5% Nonidet P-40) was added to the pellet in a 2:1 ratio (v/v) on ice for 10 min. Solution C (solution Aþ 10% glycerol and 400 mM KCl) was also added to the pellet in a 2 : 1 ratio (v/v) and mixed on ice for 20 min. The cells were then centrifuged at 15,000 g for 7 min, and the supernatant containing the nuclear extract was collected in a cold Eppendorf tube. The nuclear extracts from mouse brains were obtained using the same method. STAT3 (5’-GAT CCT TCT GGG AAT TCC TAG ATC-3, Santa Cruz Biotechnology) was labeled with T4 polynucleotide kinase and (γ-32P) ATP for 10 min at 37°C. Labeled-STAT3 was mixed with 1 μl (50,000 to 200,000 cpm) of ^32^P-labeled oligonucleotide for 20 min at room temperature. Subsequently, 1 μl of gel loading buffer was added and loaded onto a 4% non denaturing gel running until the dye reached 75% of the way down. The gel was then dried at 80°C for 2 h and exposed to film overnight at −70°C. The density of the protein was scanned and quantified by MyImage (SLB, Seoul, Korea) and Labworks 4.0 software (UVP Inc., Upland, CA), respectively.

### Pull down assay

KRICT-9 was combined with cyanogen bromide (CNBr)-activated Sepharose 4B (Sigma). Coupling buffer (0.1 M NaHCO3 and 0.5 M NaCl, pH 6.0) was used to dissolve KRICT-9 (1 mg). CNBr-activated Sepharose 4B was washed in 1 mM HCl with coupling buffer on a sintered glass filter. Then it was added to KRICT-9 with same buffer at 4°C for 24 h. Conjugated KRICT-9 with CNBr-activated Sepharose 4B was washed with three cycles of alternating pH wash buffers (buffer 1, 0.1 M acetate and 0.5 M NaCl, pH 4.0; buffer 2, 0.1 M Tris HCl and 0.5 M NaCl, pH 8.0). Conjugated KRICT-9 was then equilibrated with binding buffer (0.05 M Tris HCl and 0.15 M NaCl, pH 7.5). The control group, which was not combined with CNBr-activated Sepharose 4B beads, was prepared in the same way. KRICT-9-conjugated Sepharose 4B was mixed with the cell lysates or STAT3 recombinant protein (Abnova, Taipei, Taiwan) at 4°C for 24 h and washed three times with TBST. The bound proteins were diluted with SDS loading buffer and resolved by SDS-PAGE followed by immunoblotting with STAT3 antibodies (1:1000 dilution, Santa Cruz Biotechnology).

### Measurement of Aβ levels

Cell lysates (the same preparation of lysates as used for western blotting) were obtained using protein extraction buffer, which consists of protease inhibitor and 4-(2-aminoethyl)-benzene sulfonyl fluoride. To determinate Aβ_1-42_ levels, ELISA was performed (CUSABIO, WUHAN HYAMEI BIOTECH CO., LTD., Maryland, US). 100 μg of sample was added to pre-coated plates and incubated for 2 h at 37°C. 100 μg of labeled antibody was added for 1 h at 37°C. The samples were washed two times with washing buffer. HRP-avidin conjugate (1X) was added at 37°C and then washed after 1 h five times with washing buffer. 90μg of TMB substrate was put in the dark for 15-30 min at 37°C and 50 μg stop solution were then added. The Aβ_1-42_ levels were quantified by measuring absorbance at 450 nm using a microplate absorbance reader (Sunrise, TECAN, Switzerland).

### β-Secretase activity

We used β-secretase activity kit (Abcam) to measure β-secretase activity in astrocytes and microglial BV-2 cells. Both cells were homogenized with ice-cold extraction buffer for 10 min and then centrifuged. The supernatant was transferred to a new tube and mixed with 50 μl lysate (25-200 g of total protein), 2 × Reaction Buffer, and 2 μl β-Secretase substrate. The mixture was then placed in a covered plate and incubated in the dark at 37°C for 1 h. In order to detect the activity of β-Secretase, fluorescence was measured using a Fluostar galaxy fluorometer (excitation at 335 nm and emission at 495 nm) and Felix software (BMG Lab technologies).

### Data analysis

The data were analyzed with the Graph Pad Prism 4 ver. 4. 03 software (Graph Pad Software, La Jolla, CA) and presented as mean ± SD. Differences between groups were assessed by one-way analysis of variance (ANOVA). When the *p* value in the ANOVA test showed statistical significance (p < 0.05), the differences were evaluated using Dunnett’s test.

## SUPPLEMENTARY MATERIALS FIGURES


