# Supplementary material for: KRICT-9 inhibits neuroinflammation, amyloidogenesis and memory loss in Alzheimer’s disease models
Source: Oncotarget. 2017 Aug 2;8(40):68654–67. doi: 10.18632/oncotarget.19818 (PMC5620285; doi:10.18632/oncotarget.19818)
Supplement: Supplementary file 1 [file oncotarget-08-68654-s001.pdf]

KRICT-9 inhibits neuroinflammation, amyloidogenesis and memory loss in Alzheimer's disease models

SUPPLEMENTARY MATERIALS

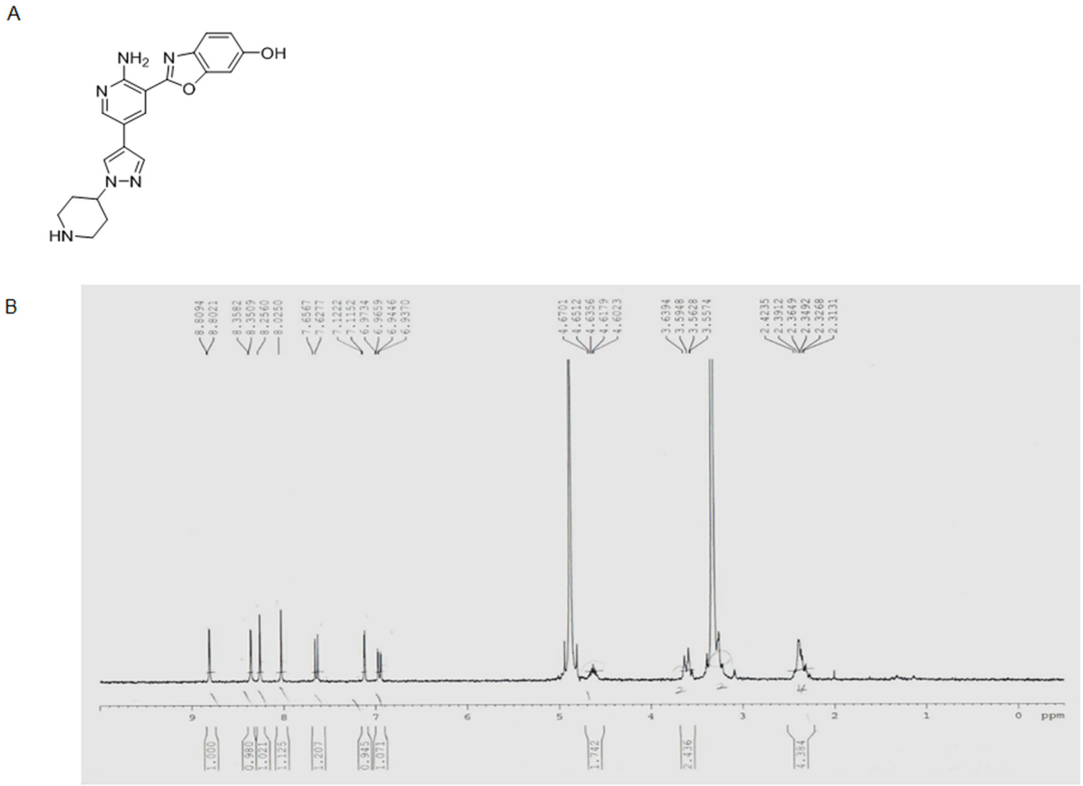

Supplementary Figure 1: Chemical structure and ^{1}H NMR of KRICT-9.

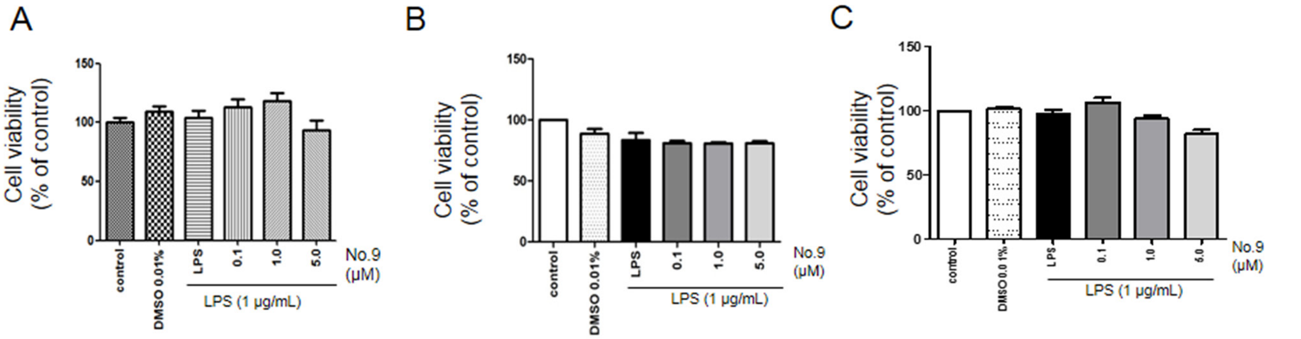

Supplementary Figure 2: Effects of KRICT-9 on the viability of RAW 264.7 cells, microglial BV-2 cells, and astrocytes. **(A)** MTT assay showing cell viability of RAW 264.7 cells, **(B)** Microglial BV-2 cells, and **(C)** astrocytes treated with 1 μg/mL of LPS alone, or with LPS plus different concentrations (0.1, 1, 5 μM) of KRICT-9, at 37 °C for 24 h. Results are given as a percentage of viable cells related to untreated controls. The data are represented asmean ± S.D for three independent experiments performed in triplicate.

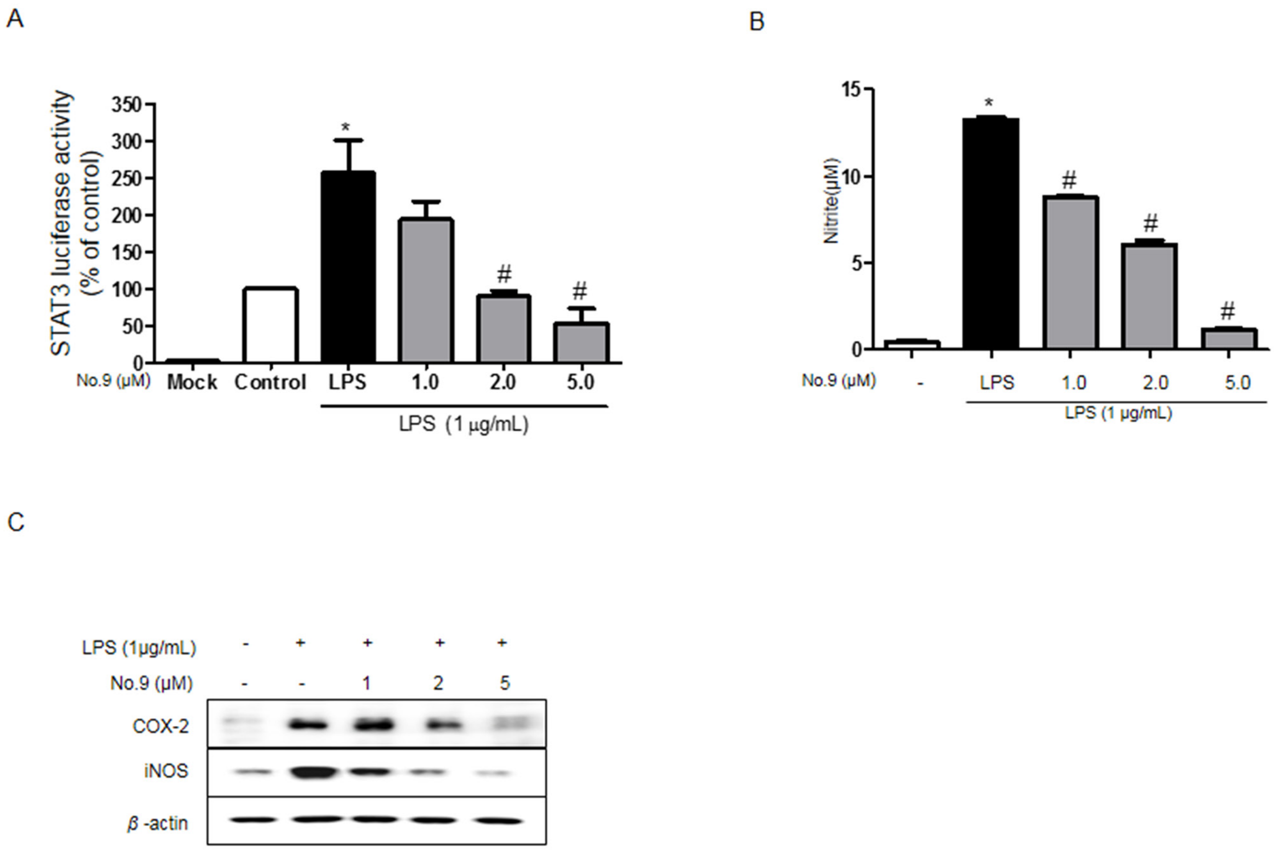

Supplementary Figure 3: KRICT-9 inhibits LPS-induced STAT3 luciferase activity, NO release, and the protein expressions of iNOS and COX-2 in RAW 264.7 cells. **(A)** Luciferase activity assay. **(B)** Griess reaction showing NO levels in the supernatant collected from RAW 264.7 cells. **(C)** Western blot using specific antibodies to show the expression of iNOS and COX-2 (β-actin was used as an internal control). Values are reported as mean ± S.D. for three experiments performed in triplicate. * Comparison against controls ($p < 0.05$), and # against LPS ($p < 0.05$).
